# Can high school students teach their peers high quality cardiopulmonary resuscitation (CPR)?

**DOI:** 10.1016/j.resplu.2022.100250

**Published:** 2022-05-24

**Authors:** Daniel Amundsen Damvall, Tonje Søraas Birkenes, Kjetil Nilsen, Solveig Haukås Haaland, Helge Myklebust, Trond Nordseth

**Affiliations:** a Faculty of Medicine and Health Sciences, Norwegian University of Science and Technology, NO-7491 Trondheim, Norway; b Laerdal Medical AS, NO-4002 Stavanger, Norway; c Nordland Hospital Trust, NO-8092 Bodø, Norway; d Department of Circulation and Medical Imaging, Faculty of Medicine and Health Sciences, Norwegian University of Science and Technology, NO-7491 Trondheim, Norway; e Department of Anesthesia and Intensive Care Medicine, St. Olav Hospital, NO-7006 Trondheim, Norway

**Keywords:** Cardiopulmonary resuscitation, CPR training, Cardiac arrest

## Abstract

**Background:**

If adolescents can teach each other cardiopulmonary resuscitation (CPR) during school hours, this may be a cost-effective approach to CPR training. The aim of this study was to evaluate CPR quality among students trained by student instructors in CPR.

**Material and methods:**

Three high schools participated. Recruited student instructors (SIs) were given a two-day course by professional instructors. Theoretic knowledge was acquired through an e-learning program. The SIs then trained fellow students in a 90-minute practical CPR session during physical education classes. All participants performed a 4-minutes test of CPR performance. Data was collected using Little Anne QCPR manikins with QCPR classroom software (Laerdal Medical Inc, Norway). Statistical equivalence in CPR performance was assessed applying the two one-sided tests (TOST)-procedure.

**Results:**

Eight professional instructors trained 76 SIs who trained approximately 2650 students in CPR. The number of available tests for analysis of student performance was 982. The compression rates were within guideline recommendations for SIs (mean 110.6, SD 5.4) and students (mean 118.6, SD 8.6). The corresponding numbers for mean compression depth were 7.2 cm (SD 0.7) and 7 cm (SD 1.0). Students demonstrated greater variation in mouth-to-mouth (MTM) skills, with only 41% performing at least 15 successful ventilations during the test. Except for the total number of MTM ventilations (mean difference −5.6), CPR performance was deemed statistically equivalent between professional instructors, SIs and students.

**Conclusions:**

High school students can be trained as CPR instructors and teach fellow students CPR with good quality, with some variation in MTM-ventilation skills.

## Introduction

In out-of-hospital cardiac arrest (OHCA), the early start of bystander cardiopulmonary resuscitation (CPR) and early defibrillation improve the chance of survival.1., 2. Bystander CPR may increase the probability of survival by two to fourfold.3., 4. Mouth-to-mouth (MTM) training amongst school children was studied in Norway as early as 1961, and chest compressions was recommended for lay people by the American Heart Association in 1974.5., 6. An increased focus on standardization of CPR training has resulted in the European Resuscitation Council (ERC) publishing guidelines on CPR training for lay people.7., 8. Weaver and et al. explored to what degree skills were retained after CPR training and found a significant decrease over time.^9^ They suggested more practical training to reduce the observed decrease in such skills, and a review of 35 studies revealed that retention of CPR skills was poor in both professionals and lay people.^10^ Both clinical and simulation studies show varying quality of CPR provided by bystanders, although this can be improved with dispatcher guidance.^11^ The ERC’s ‘KIDS SAVE LIVES’ program recommends that CPR education should be implemented in all levels of the educational system. A systematic review has evaluated several educational models that can be applied to train children in CPR, and regions implementing training for school children over several years has experienced improved outcomes after cardiac arrest.^12^ If this type of training can be offered during adolescence, the time span for which these important skills would be available for society would increase. CPR feedback technology in training manikins (QCPR, Laerdal Medical Inc., Stavanger, Norway) has been successfully applied in high-school CPR-training classes in Japan and South Korea.13., 14. Access to qualified CPR instructors in schools may be limited by costs and availability. However, if high school students were trained as CPR-instructors to teach fellow students, this could represent a low-cost option. A randomized controlled trial (RCT) of peer- versus professional led CPR training in school children demonstrated similar skills.^15^

The main aim of this study was to assess whether high-school students could be trained as CPR-instructors and teach their peers CPR with acceptable quality during physical education (PE) classes. This by assessing to what degree the instructors and students performed according to ERC guidelines for adult resuscitation, and whether the CPR provided could be considered equivalent between professional instructors, student instructors (SIs) and students.^16^

## Material and methods

The study was conducted at Bryne, Sandnes and St. Svithun high schools in Rogaland County in Norway. Data were collected as part of a volunteer educational project led by the Norwegian Resuscitation Council (NRC), supported financially by the Gjensidige Foundation (Oslo, Norway) and endorsed by the political leadership in the county. Participation was voluntary for schools and students. As the study would only collect anonymous data on CPR performance on manikins, it fell outside the working area of the Norwegian Health Research Act and formal approval by a Regional Ethical Committee was not needed. The study was given institutional approval by university leadership and registered at the Norwegian Centre for Research Data (NCRD, reference number 337048). All data were handled according to the EU GDPR-regulation. Both professional instructors and SIs gave informed consent for participation in the study, and for SIs under 18 years of age parental consent was given. The students receiving training were informed that research would be performed on anonymous data, according to NCRD recommendations.

At Bryne high school, a group of students was selected based on an interest to become CPR-instructors. At Sandnes and St. Svithun high schools, the students were selected from the Sports Education classes. The recruited SIs received a two-day training course by professional instructors from the NRC, consisting of theoretic knowledge and practical skill training to prepare them for their role as CPR-instructors. Each professional instructor trained eight SIs. An interim data analysis demonstrated some variation in the performed MTM skills at Bryne high school. For this reason, the training at Sandnes and St.Svithun high schools had more emphasis on how to provide MTM. Theoretic knowledge was provided to students and SIs through an e-learning course developed by the NRC and Laerdal Medical Inc.

The SIs hosted a 90-minute practical CPR training during PE-classes for their peer students. The course schedule is presented in Table 1. Groups of approximately eight students were trained by either one or two SIs. The course included basic theory provided by SIs and the skills needed to assess an unconscious person, to perform high quality CPR and to use an automatic external defibrillator (AED). The training program was completed between September 2018 and December 2019. The manikins and training equipment were provided by the non-profit organization ‘Frilager‘, in collaboration with the Norwegian Scout Association.^17^ ‘Frilager’ provided the equipment for the CPR training, including delivery, pick-up, and maintenance. At the end of each course, all students performed a 4-minutes test consisting of performing continuous 30:2 CPR alone. The professional instructors and SIs performed the same test. The manikin used during training was also used when performing the test. A random sample of students at Bryne high school were re-tested in the same manner in April 2019, approximately six months after their initial training, to assess skill retention. The students participated in groups of 8–25 students in the same room when performing the tests and re-tests.

**Table 1:** Timetable for the 90-minute course concept in CPR training provided by the Norwegian Resuscitation Council.

**Contents**	**Time (minutes)**
**Introduction** Equipment. The chain of survival	10
**Practical session 1** Recognition of cardiac arrest Training on recovery position	15
**Practical session 2** Compression technique and -rate.	15
**Practical session 3** How to open airway Mouth-to-mouth ventilations	5
**Practical session 4** CPR one-saver technique Communicating with dispatch	10
**Practical session 5** How to use the defibrillator Electrodes and placement	15
**Practical session 6** Simulation training CPR – defibrillation – CPR – defibrillation	15
**Summary and questions**	5
**Sum**	**90**

## Data collection

During the practical course and test, ‘Little Anne QCPR’ manikins with QCPR classroom software (Laerdal Medical Inc., Norway) was applied to collect data on CPR quality. To guide CPR feedback during training, CPR quality data was visualized in a feedback device (SkillGuide®, Laerdal Medical Inc) connected to each manikin to provide individual feedback. Feedback was not available during the 4-minutes tests. Data from manikins were collected using a tablet application (‘QCPR Classroom’ application, Laerdal Medical Inc.). All data were anonymously transferred to a cloud system and identified via the unique training manikin serial number and the date and time of CPR training. The types of data collected are demonstrated in Supplementary Table 1. The software used at Bryne high school was an early version of the software. When CPR training were provided in two adjacent classrooms, the tablet sometimes registered data from a manikin in the adjacent classroom because the manikin was within signal range of the tablet. This resulted in some cases in mixing of data in the tablet. If the participants had long pauses between compressions, a new session was created which resulted in difficulties identifying the correct dataset for some CPR tests. Several steps were taken during the data handling process to exclude these data sets. An overview of reasons for such exclusion is given in Supplementary Table 2. For the tests performed at Sandnes and Svithun high schools, the software and link-solution had been updated to reduce these problems. As exclusions were mainly due to technical problems, the tests included at Bryne were considered to be random samples representative of the tests performed and thus included in the analysis.

### Statistical analysis

The data provided by the QCPR Classrooom solution was analyzed applying Matlab version 2018b (The Mathworks, Natick, MA, USA), and further analyzed in R version 4.0.3.^18^ The R-packages ‘ggplot2’, ‘TOSTER’ and ‘wesanderson’ were applied. The proportion of students meeting the guideline recommendations was assessed applying descriptive statistics. Descriptive data are presented as means with standard deviations (SD) or proportions, as appropriate. The TOST procedure (‘two one-sided tests’) was applied to assess whether performance could be deemed statistically equivalent between professional instructor, SI and students, with the α-level set at 0.05.^19^ This provides 90% confidence intervals (CI) for differences that can be considered statistically equivalent. For compression rate and -depth we considered a difference of ±10 compressions per minute and ±10 mm in depth to be equivalent between levels of competence. This corresponds to a 10% and 20% difference in “optimal” compression quality of 100–120 compressions per minute and a compression depth of 50–60 mm, respectively. The expected number of possible successful ventilations in the 4-minute test period was 20 and 24 ventilations, depending on whether the mean compression rate was 100 or 120 per minute, respectively. We considered a difference of five successful MTM-ventilations given during the 4-minutes test to be equivalent, corresponding to a 20–25% difference compared to “optimal” (i.e. more than 15 successful MTM-ventilations given).

## Results

**Fig. 1:**
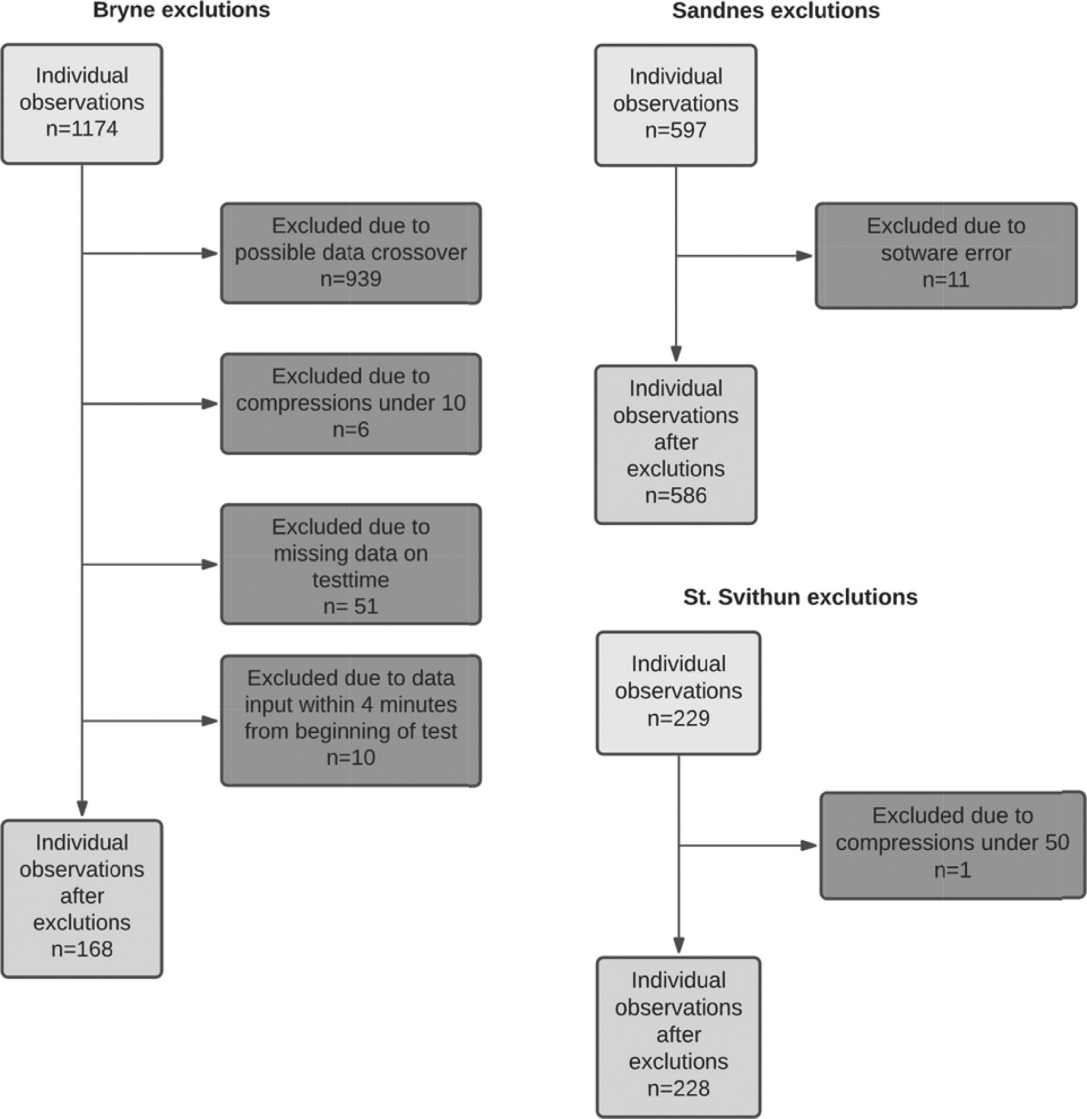
Exclusions of 4-minute CPR test for students based on the criteria given in Supplementary Table 1.

Seventy-six students received training as SIs by eight professional instructors. In the educational program, the SIs then trained approximately 1400 students at Bryne high school, approximately 1000 students at Sandnes high school and approximately 250 students at St. Svithun high school during PE-classes. Among these, 1174 students from Bryne, 597 students from Sandnes and 229 students from St. Svithun performed the 4-minute test of CPR skills at the end of the training session. Among the number of tests available for analysis, there were 982 tests from students, 76 tests from SIs and 13 tests from professional instructors. A flowchart of exclusions of student tests is demonstrated in Fig. 1.

**Fig. 2:**
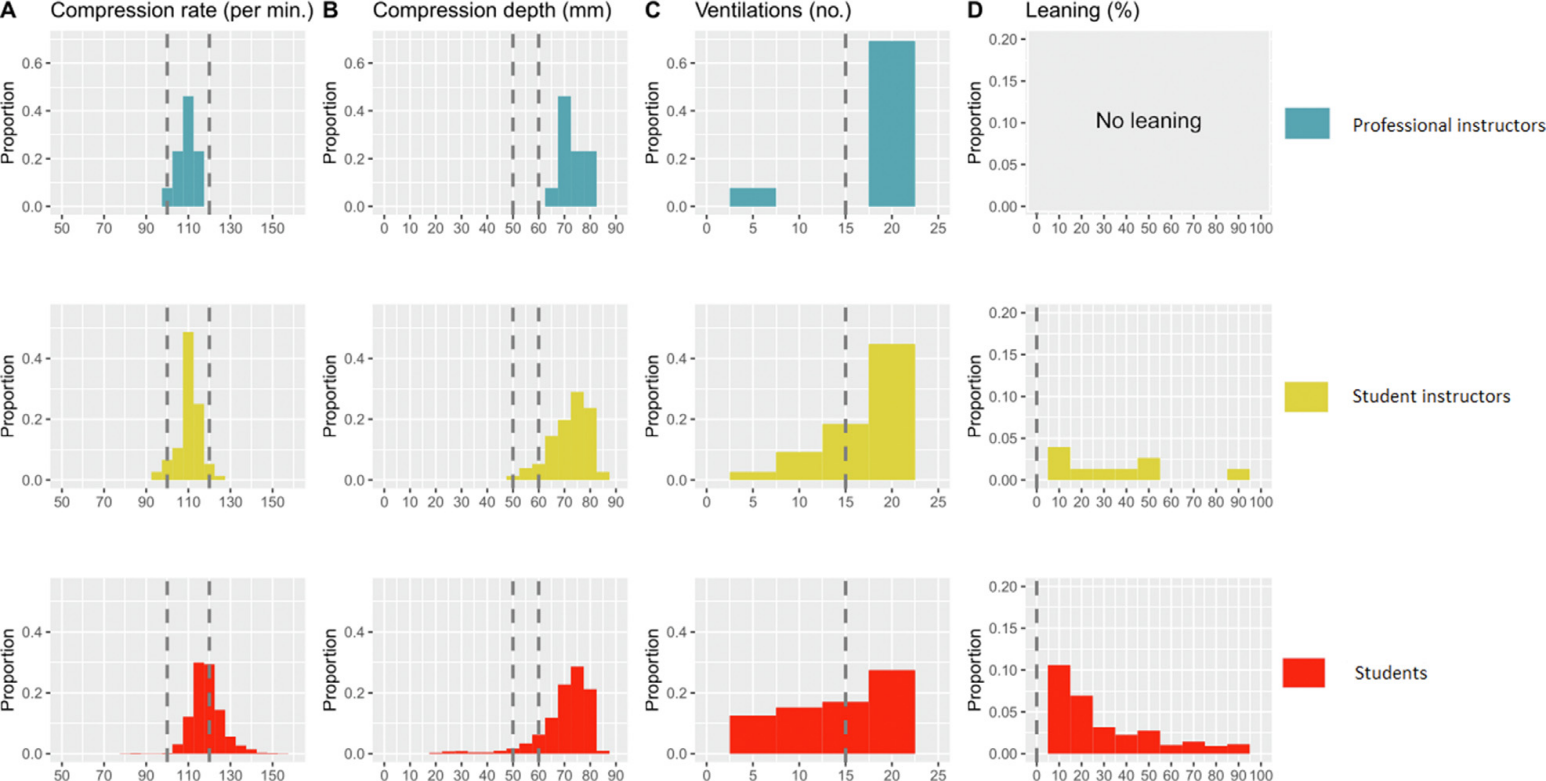
Distribution of CPR quality measures between professional instructors (blue), student instructors (yellow) and students (red).

Measures of CPR quality are reported in Table 2, stratified in the three levels of study participants. The compression rates were within guideline recommendations for professional instructors (mean 109.1, SD 4.9), SIs (mean 110.6, SD 5.4) and students (mean 118.6, SD 8.6) during the test. Students performed less successful MTM ventilations (mean 11.1, SD 8.1). The number of students and SIs that successfully performed 15 or more ventilations during the test were 41% and 73.7%, respectively. The corresponding distribution of compression rate, compression depth, number of adequate MTM-ventilations given and the proportion of “leaning” during the 4-minutes test is demonstrated in Fig. 2. Most students performed compressions with a compression depth of more than 5 cm.

**Table 2:** Quality of CPR measures between the three groups of study participants. (A) Results from the 4-minutes tests at the end of the training program. (B) Results from 4-minutes re-tests of a random sample of student instructors and students at Bryne high school, six months after the initial training. MTM = mouth-to-mouth; SD = standard deviation.

	**(A) Tests**						**(B) Re-tests**			
	**Main instructors**		**Student instructors**		**Students**		**Student instructors**		**Students**	
	**(n = 13)**		**(n = 76)**		**(n = 982)**		**(n = 11)**		**(n = 248)**	
Compression rate [min^−1^], mean (SD)	**109.1**	(4.9)	**110.6**	(5.4)	**118.6**	(8.6)	**105.5**	(5.4)	**108.4**	(12.7)
Compression depth [mm], mean (SD)	**73.0**	(4.2)	**72.1**	(7.1)	**70.4**	(10.1)	**74.0**	(5.4)	**74.3**	(7.0)
Total number of compressions, mean (SD)	**332.2**	(12.6)	**318.8**	(27.7)	**315.3**	(42.1)	**257.8**	(26.9)	**277.3**	(44.3)
Number of compressions with incomplete release (“leaning”), mean (SD)	**0**	(0)	**14.0**	(43.3)	**30.9**	(61.7)	**16.1**	(29.1)	**23.1**	(59.8)
Total number of MTM-ventilations, mean (SD)	**19.7**	(5.3)	**16.7**	(6.9)	**11.1**	(8.1)	**12.2**	(7.3)	**7.7**	(6.5)
Number of MTM-ventilations with too high volumes, mean (SD)	**4.5**	(6.2)	**2.2**	(4.7)	**2.3**	(5.6)	**0**	(0)	**0.4**	(2)

The results of the TOST tests for equivalence for the initial tests are demonstrated in Supplementary Fig. 1. The SIs demonstrated mean compression rates and -depths that were equivalent to professional instructors. The difference between students SIs and students for compression rate (mean difference 8.0, 90% CI 6.9 to 9.1) and compression depth (mean difference −1.7, 90% CI −3.1 to −0.2) was deemed equivalent within the bounds specified. The difference between students and SIs for the total number of ventilations given (mean difference −5.6, 90% CI −6.9 to −4.2), was not found to be within the defined equivalence bounds of ±5 ventilations.

## Re-tests

**Supplementary Fig. 2:**
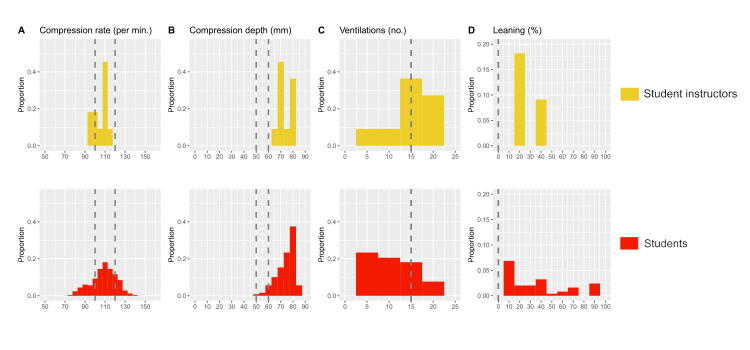
Distribution of CPR quality measures at re-testing after six months, between student instructors (yellow) and students (red).

At Bryne high school, 261 students were re-tested approximately six months after their initial training. Two tests were excluded due to technical problems, with 259 re-tests available for analysis. The compression rate was lower for both SIs (mean 105.5, SD 5.4) and students (mean 108.4, SD 12.7) compared to initial training. The number of students and SIs that successfully performed 15 or more ventilations during the re-test were 18.6% and 45.4%, respectively. Results and distributions of CPR quality measures for the re-tests are demonstrated in Table 2 and Supplementary Fig. 2.

## Discussion

In the presented “cascade model” for high school CPR training, eight professional instructors trained 76 SIs who trained approximately 2650 peer students during school hours, applying e-learning for provision of cognitive skills. An important factor for success was that equipment was delivered and maintained by an external volunteer organization, to ease logistics for the schools involved. We believe that this represents a time- and cost-efficient educational model that may ease large-scale implementation of CPR training. The skills required to give chest compression of sufficient rate and depth was well transferred between all three levels of participants in the educational model. Unfortunately, we were only able to use test data from 982 students due to technical problems, which corresponds to 37% of all students trained. After being trained by fellow students, study participants performed within ERC guidelines for both mean compression rate and minimum compression depth. However, fewer students were able to perform adequate MTM-ventilations. The high number of students performing chest compressions within guidelines recommendations is encouraging, and use of feedback devices may have further improved this. However, the observed differences should be of less clinically importance when performing real life CPR. The relatively short duration of the test may not reflect the fatigue experienced during real life CPR. However, the test was performed at the end of a 90-minute CPR training session, which may also affect results. Although rate and depth were mainly within guidelines, proper MTM-ventilations seemed harder to master with a greater variation in skills between professional instructors and students.

Lorem et al. evaluated a similar educational model using high school student as CPR-instructors for their peers and family members.^20^ The study applied an instructional video and nearly half of the persons trained were over 50 years of age. The educational model presented is also a way to increase the number of certified CPR instructors who can continue to teach CPR after they have finished high school. However, the model has some limitations and comes with some costs. The model requires school administration to be willing to invest in time and resources to initiate the training and provide follow-up. Time must be prioritized in class schedules to enable widespread participating. The SIs may need to skip their own classes to provide training for others, and we found Sport Education classes to be more flexible with respect to this. In our model, the equipment was not owned by the schools, but provided by a volunteer organization, reducing complexity and cost for the schools. Thus, all the schools utilized the same manikins and other equipment. In the Norwegian school system, CPR is embodied in the curriculum of physical education as a rather unspecific learning objective.^21^ By 10th grade the students are expected to “understand and be able to apply lifesaving first aid”.^22^ By the end of the first year of high school the objective is to “apply basic first aid”.^23^ Our model might be the start to reduce the threshold for standardized CPR training as a mandatory part of education, and thereby increasing the number of bystanders being capable of performing CPR.

The greatest variation in CPR performance was observed for MTM-ventilations. Less than 10 minutes of the course was allocated to teach this technique and our findings suggest that more time should be spent on this. Sherif et al. demonstrated that 14-year-old children could give MTM-ventilations with sufficient volume after a 15-minute theoretical lecture on this topic and a 15-minute practical demonstrations.^24^ Kim et al. demonstrated that two thirds of the high school studies performed ventilations resulting in chest rise after a 50-minute training program, although not specifying how much time was used on MTM training.^25^ In a study by Paal et al, two thirds of adolescents were able to provide MTM-ventilations with a volume higher than 700 ml after 10 minutes of training.^26^ A study on CPR quality in the elderly, with dispatcher assistance, indicated that quality of MTM-ventilations increased when instructions were given.^27^ Thus, good MTM-ventilations given by lay people is possible, but likely needs more attention during training. Moore et al. demonstrated good skill retention in MTM-ventilations even five years after practical training.^28^

It is important to note that the professional instructors had vast CPR experience, the SIs received a two-day course and the students received a 90-minute course. However, the studies by Sherif, Kim and Paal et al. demonstrate that even short CPR courses may provide sufficient CPR skills.24., 25., 26. We do not know the experience of CPR in the student group prior to this study, but we find it reasonable to assume that for the majority it was limited. Incomplete release of chest compressions (“leaning”) did not occur for the professional instructors, while it increased when SIs and students performed CPR. However previous studies have shown that leaning is easy to correct during CPR training and during dispatcher guidance.14., 29. Earlier studies have demonstrated that skill retention may vary between a few weeks and up to 12 months between initial training and re-testing 30., 31.. The students had poor skill retention with respect to MTM-ventilations with only 18.6% performing 15 or more successful MTM-ventilations. Even though skills are reduced with time, and students perform poorer after 6 months, any CPR training increases the likelihood of CPR being provided by bystanders, and the likelihood is increased if trained within the last five years.^32^

## Limitations

We were not able to collect test data on all students participating in the Educational program. Although only 37% of the tests performed by students were included in the final analysis, most of the excluded cases were from one site. As the main reason for excluding the tests were related to technical issues, we believe that the observed values represent a valid sample, that is the excluded cases could be considered ‘missing completely at random’ (MCAR). We were not able to test how well the participants applied an AED during training, although this was also a part of the educational program. Previous knowledge or training in CPR amongst the participants was not assessed. Some artificial lungs in the manikins were discovered to be partially or fully broken during training, but this was considered to concern very few manikins when performing the data analysis. However, this may have affected the registrations and the results regarding MTM performance. As we tested students in groups, they may have affected each other’s performance as compressions on manikins creates a clicking sound, and some students may have adjusted their compression rate accordingly. Too shallow compressions will not generate this sound effect, but we do not know if this may have affected the depth of compressions given. The bounds defined for equivalence were defined during drafting of the manuscript and we believe these to be consistent with what can be considered clinically relevant differences.

## Conclusion

We found that high-school students could be trained as CPR-instructors and teach their peers to perform chest compressions with acceptable quality. MTM-ventilation skills were harder to learn. If this type of model is applied in large scale CPR training in high schools, more emphasis should be put into teaching and practicing giving proper MTM-ventilations and avoid leaning.

## Conflict of interests

Author Daniel Damvall has no conflict of interest to report. Authors Trond Nordseth and Kjetil Nilsen have unpaid positions as Chairman and Deputy Chairman of the Norwegian Resuscitation Council, respectively. They have no economic conflicts of interests to report. Authors Tonje Søraas Birkenes, Solveig Haukås Haaland and Helge Myklebust are employees of Laerdal Medical Inc. The study was funded by 70 000 NOK in research grants from Laerdal Medical (Stavanger, Norway). The educational project was funded with 300 000 NOK by the Gjensidige Foundation (Oslo, Norway).

## CRediT authorship contribution statement

**Daniel Amundsen Damvall:** Conceptualization, Data curation, Methodology, Software, Investigation, Writing – original draft, Writing – review & editing, Visualization, Formal analysis. **Tonje Søraas Birkenes:** Conceptualization, Data curation, Methodology, Software, Investigation, Writing – original draft, Writing – review & editing, Visualization, Formal analysis. **Kjetil Nilsen:** Writing – original draft, Investigation, Writing – review & editing, Project administration, Resources. **Solveig Haukås Haaland:** Investigation, Writing – review & editing, Formal analysis. **Helge Myklebust:** Conceptualization, Resources, Project administration, Writing – original draft, Writing – review & editing, Formal analysis. **Trond Nordseth:** Conceptualization, Methodology, Data curation, Software, Investigation, Writing – original draft, Writing – review & editing, Visualization, Supervision, Formal analysis, Project administration, Resources.

## Appendix A Supplementary material

Supplementary Fig. 1 Two one sided test (TOST) procedure to assess equivalence between groups. Plots of mean differences and 90% confidence intervals for equivalence for compression rate, compression depth and total number of ventilations during the 4-minutes test performed at the end of training.

Supplementary Table 1 Types of data collected from the manikins. Data were collected individually on each student performing the 4-minutes CPR test.

Supplementary Table 2 Reasons for exclusions of CPR tests in the data set.
