# Supplementary material for: Can high school students teach their peers high quality cardiopulmonary resuscitation (CPR)?
Source: Resusc Plus. 2022 May 24;10:100250. doi: 10.1016/j.resplu.2022.100250 (PMC9130223; doi:10.1016/j.resplu.2022.100250)
Supplement: Supplementary Fig. 1 — Two one sided test (TOST) procedure to assess equivalence between groups. Plots of mean differences and 90% confidence intervals for equivalence for compression rate, compression depth and total number of ventilations during the 4-minutes test performed at the end of training. [file mmc1.pdf]

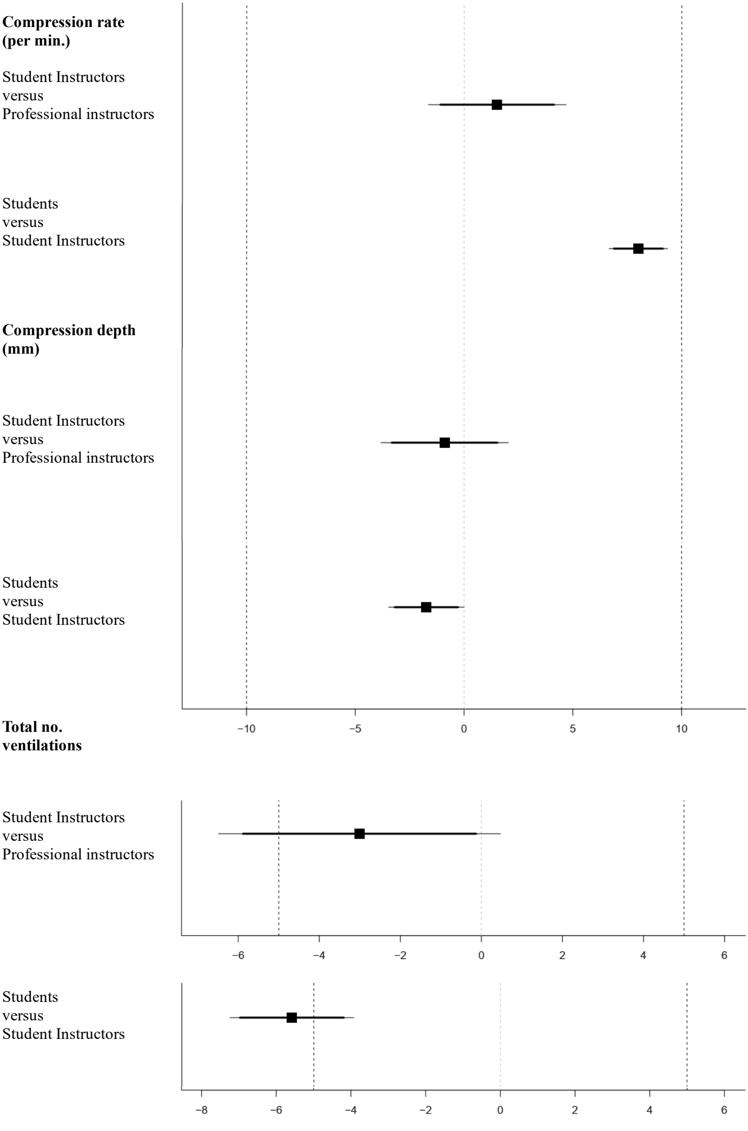

Compression rate (per min.)
Student Instructors versus Professional instructors
Students versus Student Instructors
Compression depth (mm)
Student Instructors versus Professional instructors
Students versus Student Instructors
−10
−5
0
5
10
Total no. ventilations
Student Instructors versus Professional instructors
−6
−4
−2
0
2
4
6
Students versus Student Instructors
−8
−6
−4
−2
0
2
4
6
