# Supplementary material for: Can high school students teach their peers high quality cardiopulmonary resuscitation (CPR)?
Source: Resusc Plus. 2022 May 24;10:100250. doi: 10.1016/j.resplu.2022.100250 (PMC9130223; doi:10.1016/j.resplu.2022.100250)
Supplement: Supplementary Table 1 — Types of data collected from the manikins. Data were collected individually on each student performing the 4-minutes CPR test. [file mmc2.docx]

| **CPR Training data** |
| --- |
| - - Time and duration of training |
| **Chest compressions** |
| - - Total number of compressions |
| - - Average compression rate |
| - - Average compression depth |
| - - Number of compressions with adequate depth |
| - - Number of compressions with leaning |
| - - Number of compressions with complete release |
| **Mouth to mouth ventilations** |
| - - Total number of ventilations |
| - - Number of ventilations with high volume |
| - - Number of ventilations with adequate volume |
