# Supplementary material for: Can high school students teach their peers high quality cardiopulmonary resuscitation (CPR)?
Source: Resusc Plus. 2022 May 24;10:100250. doi: 10.1016/j.resplu.2022.100250 (PMC9130223; doi:10.1016/j.resplu.2022.100250)
Supplement: Supplementary Table 2 — Reasons for exclusions of CPR tests in the data set. [file mmc3.docx]

| **Exclusions of tests at Bryne High School** |
| --- |
| Manikins with new data input within the first 4 minutes of a test start |
| Complete sets from the same day where there is obvious data crossover across both sets |
| Abnormaly large sets because of possible data collection from more than one classroom |
| Manikins in a specific set without data at the probable test time, assuming the manikin was not used during testing. |
| Manikins with number of compressions less than 10 at test time |
| Manikins with identical data input at different time stamps. |
| **Exclusion of tests at Sandnes and St.Svithun High Schools** |
| Manikins with number of compressions less than 50 at test time |
| Manikins with a negative number of compressions or ventilations, assuming software error). |
| If technical defects in the manikin were found during or after testing |
